# The complete chloroplast genome of *Oenanthe javanica*

**DOI:** 10.1080/23802359.2020.1806128

**Published:** 2020-08-12

**Authors:** Zhen Zhang, Huanze Dong, Meng Yuan, Yan Yu

**Affiliations:** a Wu yuzhang Honors College, Sichuan University, Chengdu, P. R. China; b Key Laboratory of Bio-Resources and Eco-Environment of Ministry of Education, College of Life Sciences, Sichuan University, Chengdu, P. R. China

**Keywords:** Chloroplast, genome, *Oenanthe javanica*, Oenantheae

## Abstract

*Oenanthe javanica* (Bl.) DC. (Apiaceae) is a herbaceous species naturally distributed in China. The complete chloroplast genome sequence of *Oenanthe javanica* was generated by de novo assembly using whole genome next generation sequencing data. The chloroplast genome of *Oenanthe javanica* was 154,246 bp in length and divided into four distinct regions, such as large single copy region (84,142 bp), small single copy region (17,238 bp) and a pair of inverted repeat regions (26,433 and 26,433 bp). The genome annotation predicted a total of 123 genes, including 85 protein-coding genes, 36 tRNA genes, and 8 rRNA genes. Phylogenetic analysis with the reported chloroplast genomes revealed that *Oenanthe javanica* is nested in Apiaceae tribe Oenantheae and has a close relationship to *Cicuta virosa* and *Cryptotaenia japonica.*

*Oenanthe javanica* (Bl.) DC. (Apiaceae) is an herbaceous species naturally distributed in China. It wildly grows in low-lying places or beside ponds and gutters, 600–4000 m and it is an edible vegetable with high vitamin and can be used as a medicine to lower blood pressure (Pan and Watson 2005). Although several phylogenetic studies reported transcriptome sequencing and small RNA sequencing of *Oenanthe javanica* (Jiang et al. 2015), the complete chloroplast genome sequence is not available till now. Here, we report the complete chloroplast genome sequence of *Oenanthe javanica* to provide a genomic resource and to clarify the phylogenetic relationship of this plant with other species in the Apiaceae family. Total genomic DNA was isolated from mature leaves sampled from the campus of Sichuan University, Chengdu, Sichuan, China. Voucher specimens were deposited in SZ (Sichuan University Herbarium) and the specimen number of the specimen is wk000001. Morphological characters were measured using Karyotype (Altınordu et al. 2016). Chloroplast DNA (cpDNA) was extracted by High-salt Low-pH (HSLp) method and Sucrose DNase (SucDNase) method. The isolated genomic was manufactured to average 400 bp paired-end (PE) library using Illumina Hiseq platform (Illumina, San Diego CA, USA), and sequenced by Illumina genome analyzer (Hiseq PE150). The raw reads were then assembled using NOVOPlasty 4.1 (Dierckxsens et al. 2017) with ribulose-1,5-bisphosphate carboxylase/oxygenase (rbcL) gene from *Cicuta virosa* as seed. The draft sequence obtained from NOVOPlasty was corrected manually by clean reads mapping using bowtie2 (Langmead and Salzberg 2012) and Tablet (Milne et al. 2013). The genes in chloroplast genome were predicted using Geneious (Kearse et al. 2012) and corrected manually. The complete chloroplast genome of *Oenanthe javanica* (GenBank accession no.MT622521) was a circular form of 154,246 bp in length, which was separated into four distinct regions such as large single copy (LSC) region of 84,142 bp, small single copy (SSC) region of 17,238 bp, and a pair of inverted repeat regions of 26,433 bp and 26,433 bp. Overall, GC contents of chloroplast genomes were 37.6%. The chloroplast genome contained a total of 123 genes including 86 protein-coding genes, 36 tRNA genes, and 8 rRNA genes.

To understand the phylogenetic relationship between *Oenanthe javanica* and related species, the complete chloroplast genome sequences of 29 genera (34 species) from Apiaceae were aligned by MAFFT (Katoh et al. 2002) and trimmed properly by trimAl v1.4 (Capella-Gutierrez et al. 2009). The evolutionary history was inferred by using the Neighbor-joining method in MEGA7.0 (Kumar et al. 2016). The percentage of replicate trees in which the associated taxa clustered together in the bootstrap (BS) test (10,000 replicates) are shown next to the branches (Figure 1). As was expected, *Oenanthe javanica* was placed within Apiaceae tribe Oenantheae, and comprise a clade with *Cicuta virosa* and *Cryptotaenia japonica* with 100% BS value.

**Figure 1.**
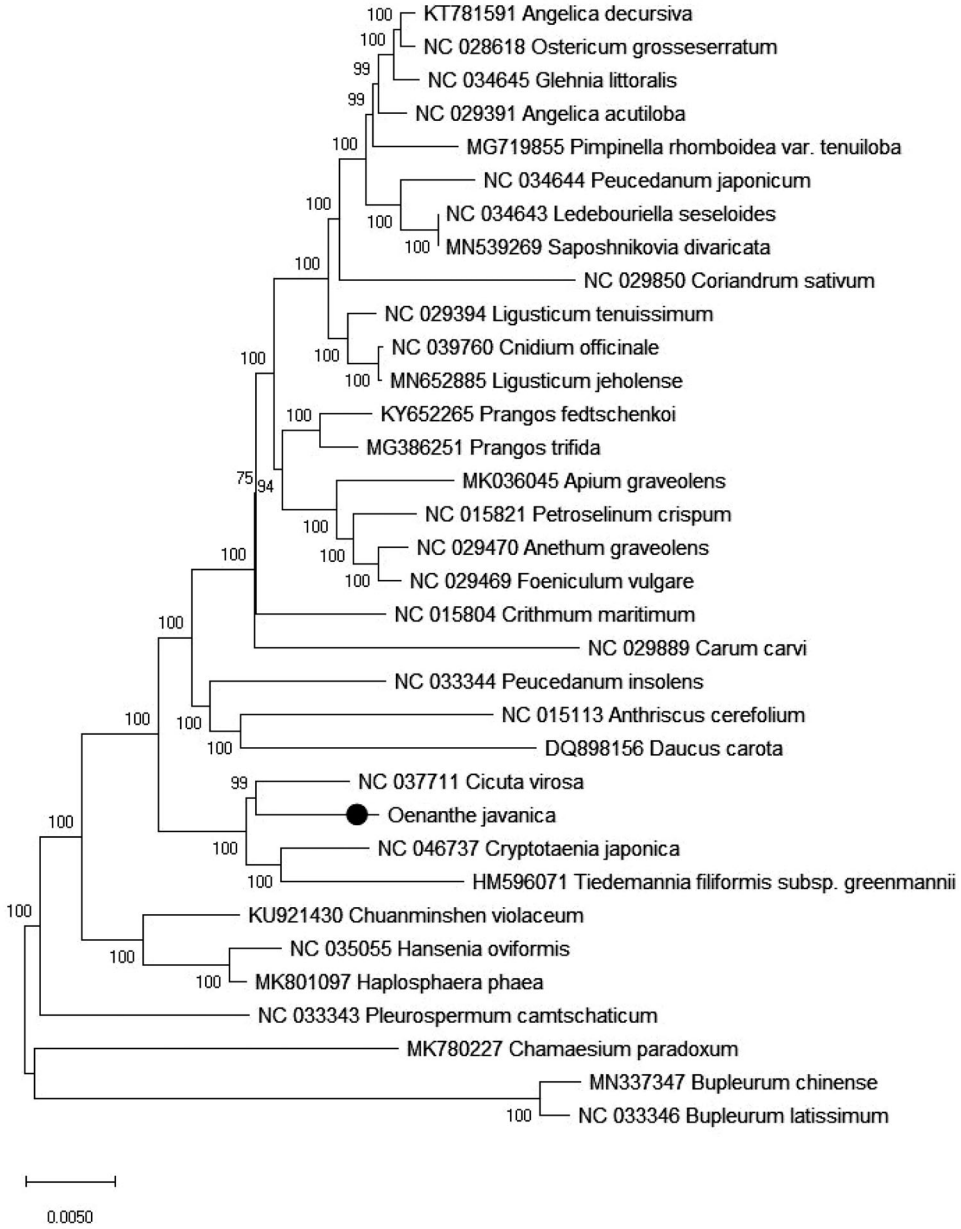
Neighbor-Joining tree of *Oenanthe javanica* and related species using chloroplast sequences. Numbers on the nodes are bootstrap values from 10000 replicates.

## Data Availability

The data that support the findings of this study are openly available in GenBank of NCBI at https://www.ncbi.nlm.nih.gov, reference number MT622521.
